# Vaccine Development Against COVID-19 Prior to Pandemic Outbreaks, Using *in vitro* Evolution and Reverse Genetics

**DOI:** 10.3389/fimmu.2020.02051

**Published:** 2020-08-14

**Authors:** Hatem Zayed

**Affiliations:** Department of Biomedical Sciences, College of Health Sciences, QU Health, Qatar University, Doha, Qatar

**Keywords:** VLP vaccines, COVID-19, pandemic outbreak, SARS-CoV2, integrating vectors, coronaviruses

The coronavirus disease 2019 (COVID-19) is caused by severe acute respiratory syndrome coronavirus 2 (SARS-CoV-2), which is an enveloped, non-segmented, positive-sense RNA virus (1). The complete genome of SARS-CoV-2 is 29.9 kb (2, 3). The virus genome contains four essential proteins that are believed to be important for the infectious ability of the virus, the glycoprotein spike (S), nucleocapsid (N), matrix (M), and small envelope (E) proteins (4). The S glycoprotein, which mediates entry of the virus into the target cells, is the main target for host defense antibodies (5).

As of May, 2020, the COVID-19 pandemic has spread to 213 countries and territories worldwide with nearly 6 million confirmed cases and ~6% mortality (who.int). As the outbreaks spread, scientists across the globe are racing to develop vaccines against COVID-19. Since coronaviruses are increasing alarmingly, there is an urgent need for a safe and effective vaccine to prevent the spread of the virus during pandemic outbreaks, and stop deaths associated with the virulent COVID-19. However, developing vaccines that are safe and effective requires a lot of time and testing. It is estimated that 18 months are needed to develop such a vaccine.

Although it is challenging to predict the severity, time, and location of future coronavirus pandemics, we can be prepared for the highly pathogenic strains that are likely to reemerge and cause future pandemics. This can be done using previous epidemiological studies on coronaviruses. For example, in 2019, Chinese scientists anticipated that there would be a potential bat coronavirus that would likely emerge and infect humans, and might cause an imminent outbreak in China (6). Unfortunately, the efforts of these Chinese scientists were met with no interest from the Chinese government, evidenced by the lack of proper preparation for the current pandemic when it appeared in China a few months ago. We now know that SARS-CoV-2 shares 88% identity with two SARS-like coronaviruses (bat-SL-CoVZXC21 and bat-SL-CoVZC45) that both originated in China, and use the same human angiotensin-converting enzyme 2 receptor for cell entry during the process of infection (3). If we had reacted to these predictions, then we would very likely have avoided the current crisis. In response to such forewarnings from scientists, a predictive vaccine could have been designed and developed for the potential virus pandemic. Developing a vaccine during or after the pandemic outbreaks is too slow to provide timely responses against COVID-19, and risks many lives. Producing an efficient and safe vaccine ready for human use can take up to 18 months, according to the World Health Organization (WHO). Therefore, anticipating the virus mutations responsible for the possible reemergence of highly pathogenic virulent strains may be a means by which to prepare for future, newly emerging, pandemic strains.

The process of preparing a predictive vaccine can be summarized as follows: (1) The SARS-CoV-2 genome would be used as a template for *in vitro* evolution through DNA shuffling techniques (7, 8). Random recombination of a viral genome in a test tube mimics the possible assortment and mutations that occur in the virus in nature, creating all possible random recombination. These changes could be incorporated into the four essential viral genes (S, N, M, and E). (2) These genes would then be subcloned individually into integrating gene delivery vehicles, such as lentiviral vectors (9) or transposons (10, 11). (3) Using reverse genetic strategies (12, 13), the recombinant constructs would be transfected into cell lines susceptible to coronaviruses (14), leading to secretion of virus-like particles (VLPs) from the cells into the culture media. (4) The recombinant VLPs could then be harvested and purified from the supernatant of the culture media. (5) VLPs would then be tested for proper assembly and integrity using electron microscopy and different methods of protein quantification. (6) All possible mutant VLPs would be tested using different functional assays to check for possible antigenicity. (7) The candidate VLPs proven to be functional and highly immunogenic could then be used in challenge experiments using animal models and recombinant live virulent viruses believed to be highly pathogenic. (8) The VLPs that are highly protective against the highly pathogenic recombinant strains would be further selected and stable cell lines made from all candidate VLP vaccines. (9) These cell lines could be expanded using bioreactors and stored for further use. Lentiviral vectors could generate a stable cell line that is transgenic for the highly immunogenic antigenic determinants of COVID-19 (15), and would be able to continuously secrete VLPs into the culture media (16). Thereafter, during the time of pandemic, suitable stored transgenic cell lines could be used, based on the reemergent pandemic viral mutant strain, and could be easily shipped across the globe, thawed, and manufactured on a large scale in customized large-sized bioreactors (Figure 1). VLP vaccines could be used as therapeutic vaccines and administered to infected individuals (17), or as vaccines into healthy non-infected individuals. The immunodominant epitopes (18) of the viral mutants specific for the virus would elicit potent immune responses that could be life-saving (19). The genomeless hollow shells would mimic the actual live virus in terms of eliciting a strong immune response; however, these shells are neither replicative nor infectious by themselves (20).

**Figure 1:**
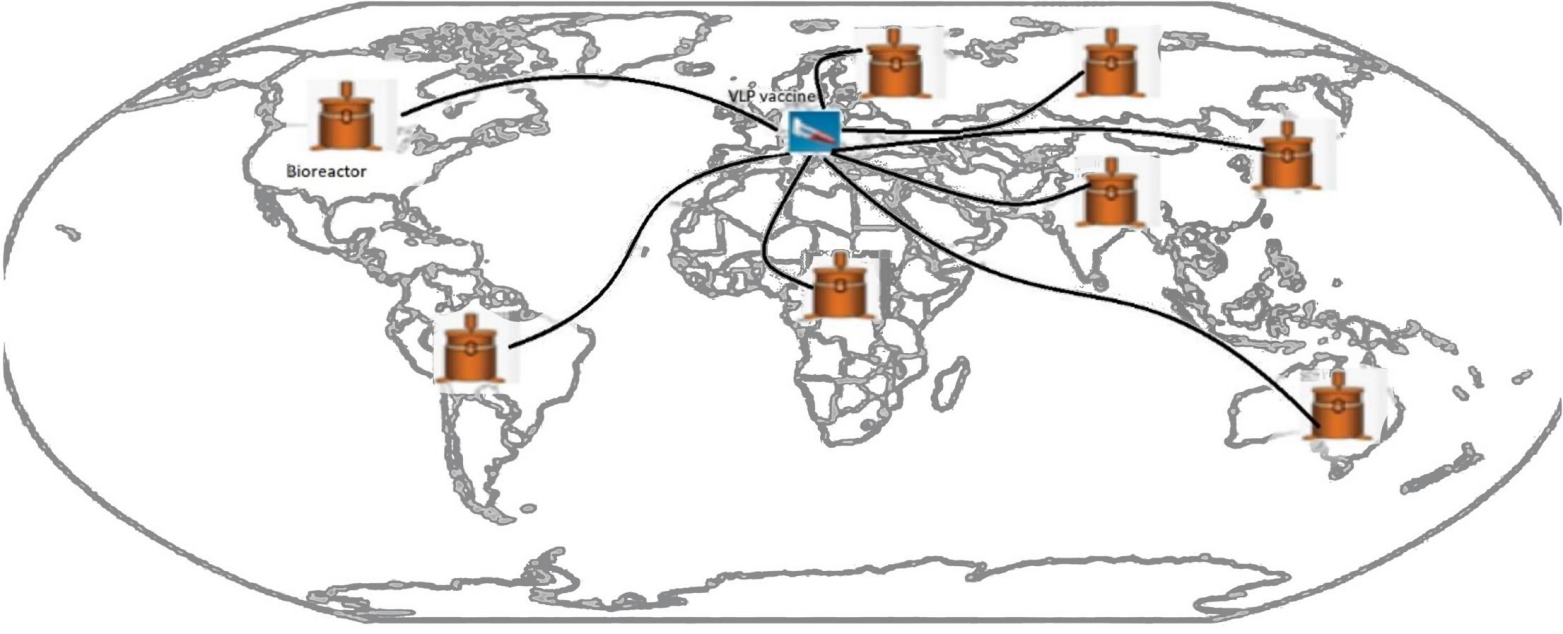
The possible satellite WHO-supervised distributions sites of the preemptive VLP vaccine during COVID-19 pandemic.

Such a project should be done through international collaborations and under the supervision of the WHO. Stocks of these VLP vaccines could be stored as vials of transgenic cell lines, able to be regularly expanded and checked for their quality and ability to generate VLP vaccines. Stocks of these vials could be kept in different countries with satellite distributors managed and administered by the WHO. This project would require scientists with high degrees of skill that are trained in the field of vaccine design and development, and trained in several other fields such as molecular biology, virology, infectious diseases, and cell biology.

The development of VLP vaccines against reemerging viral pandemics would be far affordable than the economic costs of the current COVID-19 pandemic. Such project requires concerted global efforts of multiple organizations, which is expected to save thousands of lives. I do believe that the time has come for all government officials and policymakers to listen very carefully to science and scientists' recommendations to ensure the health and well-being of people of our planet.

## Author Contributions

HZ conceptualized the study and wrote the manuscript.

## Conflict of Interest

The author declares that the research was conducted in the absence of any commercial or financial relationships that could be construed as a potential conflict of interest.

## Abbreviations

COVID-19: coronavirus disease 2019
SARS-CoV-2: severe acute respiratory syndrome coronavirus 2
VLP: virus-like particle
WHO: World Health Organization.
